# Cytokine production and signaling pathways in respiratory virus infection

**DOI:** 10.3389/fmicb.2013.00276

**Published:** 2013-09-17

**Authors:** Hirokazu Kimura, Masakazu Yoshizumi, Haruyuki Ishii, Kazunori Oishi, Akihide Ryo

**Affiliations:** ^1^Infectious Disease Surveillance Center, National Institute of Infectious DiseasesTokyo, Japan; ^2^Gunma Prefectural Institute of Public Health and Environmental SciencesGunma, Japan; ^3^Department of Molecular Biodefence Research, Graduate School of Medicine, Yokohama City UniversityKanagawa, Japan; ^4^Department of Respiratory Medicine, School of Medicine, Kyorin UniversityTokyo, Japan

**Keywords:** cytokine, signaling pathway, respiratory virus, innate immunity, virus-induced asthma

## Abstract

It has been confirmed that respiratory virus infections can induce abberant cytokine production in the host. These cytokines may be associated with both elimination of the virus and complications in the host, such as virus-induced asthma. Representative host defense mechanisms against pathogens, including bacteria and viruses, are mediated by the innate immune system. Cells of the innate immune system express essential molecules, namely pattern recognition receptors (PRRs), such as Toll-like receptors, nucleotide-binding oligomerization domain-like receptors, and retinoic acid-inducible gene-I-like receptors. These PRRs can recognize components of pathogens such as bacterial lipopolysaccharide, viral antigens, and their genomes (DNA and RNA). Furthermore, PRRs activate various signaling pathways resulting in cytokine production against pathogen infection. However, the exact mechanisms remain unknown. In this review, we mainly focus on the representative mechanisms of cytokine production through PRRs and signaling pathways due to virus infections, including respiratory virus infections. In addition, we describe the relationships between respiratory infections and virus-induced asthma.

## INTRODUCTION

Coordination between innate and adaptive immunity against pathogens is indispensable in higher organisms including humans (Medzhitov, 2007). In particular, innate immunity plays a critical role during primary infection with various bacteria and viruses (Barbalat et al., 2011; Jarchum and Pamer, 2011; Kumar et al., 2011). The specific recognition of microorganisms may represent the basis of innate immunity (Barbalat et al., 2011; Jarchum and Pamer, 2011; Kumar et al., 2011). Specific recognition systems have gradually been clarified and the common platforms are Toll-like receptors (TLRs), the NLR family (nucleotide-binding oligomerization domain-like receptors), and the RLR family [RIG (retinoic acid-inducible gene)-I-like receptors] (Kumar et al., 2011; Yu and Levine, 2011). These molecules are called pattern recognition receptors (PRRs). PRRs can recognize lipopolysaccharides (LPS), viral antigens, and bacterial/viral genomes, leading to the activation of intrinsic signaling pathways (e.g., myeloid differentiation factor 88; MyD88) and the production of various cytokines (Barbalat et al., 2011; Jarchum and Pamer, 2011; Kumar et al., 2011; Ting Tan et al., 2013). The production of such cytokines may activate leukocytes and eliminate the infective agents (Chehadeh and Alkhabbaz, 2013; Ting Tan et al., 2013).

At present, over 50 cytokines have been discovered. They form networks and play pivotal roles in infectious and allergic diseases (Barnes, 2008; Desai and Brightling, 2012; Holgate, 2012). These cytokines are mainly produced by blood cells, lymphoid tissues, and epithelial cells. For example, interferons (IFNs), which are anti-viral cytokines produced by lymphocytes and epithelial cells, are dramatically induced by various viral infections such as influenza (Qin et al., 2011b; Högner et al., 2013; Lopušná et al., 2013). This induction may contribute to the elimination of viruses *in vivo*. Indeed, we use recombinant IFNs to treat chronic viral infections such as hepatitis C (Nagao et al., 2012; Slim and Afridi, 2012). On the other hand, aberrant induction of other cytokines such as interleukin (IL)-4 may induce various allergic diseases, such as virus-induced asthma (Baraldo et al., 2012; Krishnamoorthy et al., 2012). In addition, aberrant induction and an imbalance of various proinflammatory cytokines, for example, IL-1β, IL-6, and tumor necrosis factor (TNF), may induce severe systemic inflammatory response syndrome (Watanabe et al., 2003; Xu et al., 2012). Thus, various cytokines may be associated with the pathophysiology of inflammation and remodeling of the airways post-infection.

Acute respiratory illnesses (ARI) are the most common diseases in humans. Accumulating evidence suggests that around 80% of the causative agents of ARI may be respiratory viruses (Heymann et al., 2004; Fujitsuka et al., 2011). The prognosis is good in most patients with viral ARI; however, viruses causing ARI may be responsible for more severe diseases like bronchitis, bronchiolitis, and pneumonia (Domachowske and Rosenberg, 1999; Sigurs, 2002; Kusel et al., 2007). Furthermore, representative respiratory viruses such as respiratory syncytial virus (RSV) may induce bronchiolitis or pneumonia with wheezing in infants (Stein et al., 1999; Sigurs et al., 2000).

To better understand host defense mechanisms against viruses, it is important to clarify these molecular mechanisms. In this review, we focus on cytokine production and signaling pathways during viral infection. We also discuss the relationships between cytokine profiles and virus-induced asthma under the main theme “virus-induced asthma.”

## INFECTION AND INNATE IMMUNITY

**FIGURE 1:**
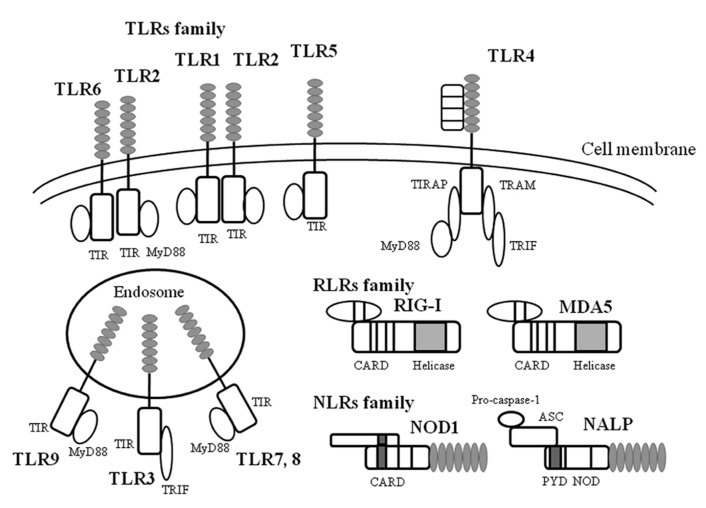
** A schematic illustration of microbial infection and innate immunity-related molecules.** TLR, RLR, NLR are all expressed on and in a variety of cells in host tissues that recognize a diverse range of microbial signatures. The ectodomain of the TLR consists of leucine-rich repeats. Upon ligand stimulation, all TLRs except TLR3 recruit the adaptor MyD88. TLR3 is activated via the adaptor protein TRIF (Toll/IL-1 receptor domain containing adaptor protein inducing interferon-1β). RLRs consist of a group of cytosolic RNA helicases. RIG-I and MDA5 are composed of two amino-terminal CARD domains and a central helicase domain. In contrast to RIG-I, MDA5 lacks the C-terminal repressor domain. NLR are composed of a central nucleotide-oligomerization domain and a C-terminal leucine-rich repeat domain involved in the recognition of pathogens. The N-terminal domain can be either a CARD or a PYD domain. The NLR subfamily is characterized by an N-terminal PYD domain. ASC, apoptosis-associated speck-like protein containing caspase recruitment domain; CARD, caspase recruit domains; MDA, melanoma differentiation-associated protein; MyD88, myeloid differentiation factor 88; NALP, NACHT, LRR and PYD domains-containing protein; NOD, nucleotide-binding oligomerization domain; PYD, pyrin domain; RIG, retinoic acid-inducible gene; TIR, Toll/IL-1 receptor-like structure as intercellular domain; TIRAP, TIR domain-containing adaptor protein; TLR, Toll like receptor; TRAM, TRIF-related adaptor molecule; TRIF, TIR domain-containing adaptor including IFN-1β.

Host defense mechanisms against microbial infections constitute the main purpose of innate immunity (an archaic term meaning natural resistance; Jarchum and Pamer, 2011; Kumar et al., 2011). The main platforms of the molecular groups against the pathogens include TLRs, the NLR family (nucleotide-binding oligomerization domain-like receptors), and the RLR family (RIG-I-like receptors). These molecules/receptors can recognize various components including LPS derived from bacteria, viruses, and fungi, viral antigens, and the pathogen genomes (Barbalat et al., 2011; Jarchum and Pamer, 2011; Kumar et al., 2011; Yu and Levine, 2011). Subsequent events activate innate immunity involved in cytokine production in the host (Barbalat et al., 2011; Kumar et al., 2011; Yu and Levine, 2011; Ting Tan et al., 2013). The innate immune system initiates a different mechanism against each pathogen (Chehadeh and Alkhabbaz, 2013; Kemp et al., 2013). Thus, these pathogen-associated receptors are called “PRRs” (Kawai and Akira, 2007; Pang and Iwasaki, 2012). Schematic illustrations of these families are shown in **Figure 1**.

## TOLL-LIKE RECEPTORS

**FIGURE 2:**
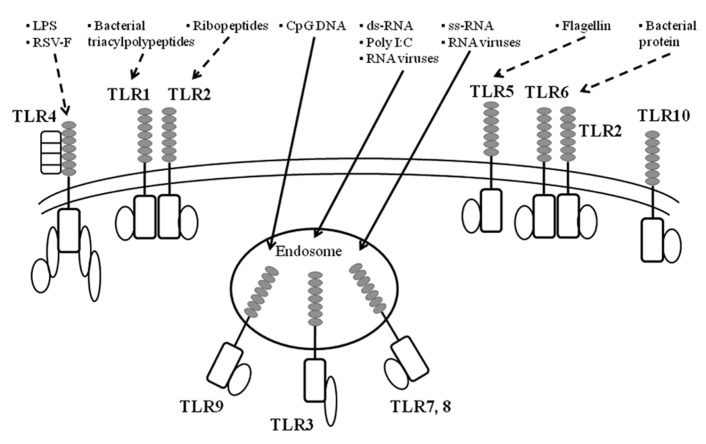
** A summarized illustration ofTLRs and their ligands.** TLR are key initiators of inflammation during host defense. Acting as dimers, the ten different TLRs display differential patterns of recognition of microbial products. TLR4 detects LPS, which is specific to Gram-negative species. TLR1/2 and TLR2/6 recognize triacylated and diacylated polypeptides, respectively, from mycobacteria. TLR5 responds to flagellin from the flagella of multiple bacteria. TLR7 and TLR8 detect single-stranded RNA (ss-RNA) from viruses, and TLR9 detects CpG DNA, which is common in bacterial and viral pathogens. TLR3 recognizes double-stranded RNA (ds-RNA) or synthetic compounds such as Poly I:C.

As already mentioned, virus infections can induce the production of various cytokines (Yoshizumi et al., 2010; Ishioka et al., 2011; Kato et al., 2011). TLRs may be responsible for cytokine production in bacteria- or virus-infected epithelial cells and immune cells (Rudd et al., 2005; Barbalat et al., 2009). In general, it is thought that TLRs play pivotal roles in innate immunity against viral and bacterial infections (Kawai and Akira, 2011; McIsaac et al., 2012). In humans, 10 types of TLRs have been identified (Akira et al., 2006; Takeuchi and Akira, 2009; Kumar et al., 2011). TLRs possess an extracellular domain containing leucine-rich repeats and a Toll/IL-1 receptor-like structure as the intercellular domain (TIR domain; Janssens and Beyaert, 2003; Akira et al., 2006). TLRs can be classified into three types: lipid ligands (TLR1, 2, 4, 6, and 10), protein ligands (TLR5), and nucleic acid ligands (TLR3 and 7–9; Janssens and Beyaert, 2003; Akira et al., 2006; Vandevenne et al., 2010). Thus, the TLR family can recognize various biological components derived from microorganisms (Janssens and Beyaert, 2003; Akira et al., 2006; Vandevenne et al., 2010). TLR1, 2, 4, 5, and 6 are transmembrane proteins (Janssens and Beyaert, 2003; Akira et al., 2006; Vandevenne et al., 2010), which mainly bind to bacterial components such as bacterial triacylpolypeptides (TLR1), ribopeptides (TLR2), LPS (TLR4), and the bacterial protein flagellin (TLR5; Janssens and Beyaert, 2003; Akira et al., 2006; Vandevenne et al., 2010). Interestingly, TLR4 proteins also bind to a major viral antigen of RSV F (fusion) protein (See and Wark, 2008; Klein Klouwenberg et al., 2009). In addition, TLR3, 7, 8, and 9 reside in the endosomes in cells (Janssens and Beyaert, 2003; Akira et al., 2006; Vandevenne et al., 2010). TLR7 and 8 can recognize single strand viral RNA molecules, while TLR3 can also recognize double strand RNA and poly I:C (polyinosinic polycytidylic acid; Janssens and Beyaert, 2003; Akira et al., 2006; Vandevenne et al., 2010). Thus, TLR3, 7, and 8 are essential receptors for many types of RNA viruses including paramyxoviruses (Sendaivirus) and orthomyxovirus (influenza viruses; Melchjorsen et al., 2005; Hammerbeck et al., 2007; Klein Klouwenberg et al., 2009). Moreover, TLR9 recognizes CpG DNA (a phosphodiester bond within cytosine and guanine; Janssens and Beyaert, 2003; Akira et al., 2006; Vandevenne et al., 2010). Thus, TLRs can bind to various components of microorganisms including viruses, leading to cytokine production through activation of signaling pathways in pathogen-infected cells (Janssens and Beyaert, 2003; Akira et al., 2006; Vandevenne et al., 2010). An illustrated summary is shown in **Figure 2**.

## TLRs-ASSOCIATED SIGNALING PATHWAYS AND CYTOKINE PRODUCTION

**FIGURE 3:**
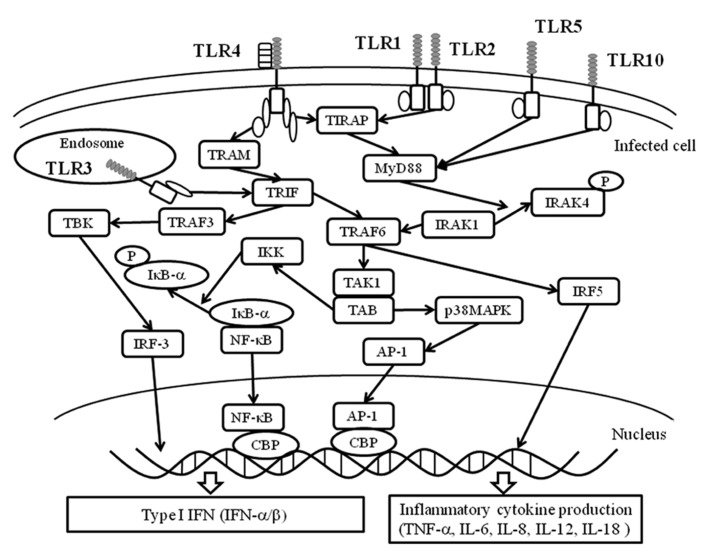
** Essential TLR-associated signaling pathways and cytokine production.** Upon ligand stimulation, all TLRs, except TLR3, recruit the adaptor MyD88. In turn, MyD88 binds to a protein complex composed of IRAK4, IRAK1, and TRAF6. TRAF6 undergoes self-polyubiquitination resulting in the phosphorylation of TAK1. In turn, TAK1 activates IKK complex that leads to the phosphorylation, ubiquitination, and degradation of IκBα. This allows NF-κB to translocate into the nucleus. Simultaneously, the TAK1-containing complex activates the p38 MAP kinase pathway triggering the activation of AP-1. Together, NF-κB and AP-1 induce the expression of pro-inflammatory cytokines. TLR4 and TLR2, in combination with TLR1 or TLR6, recruit TIRAP that serves as a link adaptor for the recruitment of MyD88. Moreover, TLR4 recruits a second link adaptor namedTRAM that allows interaction with the adaptor TRIF. Upon stimulation with an agonist, TLR3 recruits TRIF. TRIF-mediated activation of NF-κB and AP-1 also occurs through a TRAF6-dependent pathway. Upon stimulation, TRIF also binds TRAF3, which activates TBK. TBK phosphorylates IRF3 and permits its homodimerization and nuclear translocation. IRF-3, along with NF-κB and AP-1, cooperate to induce the expression of type I IFNs. AP-1, activator protein-1; CBP, cyclic AMP response element binding factor binding protein; IκB-α, inhibitor of NF-κB -α; IKK, IκB kinase complex; IRF, interferon regulatory factor; IRAK, IL-1 receptor-associated kinase; NF-κB, nuclear factor kappa light chain enhancer of activated B cells; p38MAPK, p38 mitogen activated protein kinase; TAB, TAK1 binding protein; TBK, TANK(TRAF family member NF-κB activator) -binding kinase; TAK1, TGF-1β activated kinase 1; TRAF, TNF receptor associated factor.

A summarized illustration is shown in **Figure 3**. TLRs possess a common TIR domain (Janssens and Beyaert, 2003; Akira et al., 2006). The TIR domain can bind an adaptor molecule, MyD88 (Picard et al., 2011). MyD88 triggers downstream signaling pathways such as IRAK (IL-1 receptor-associated kinase)-1/4, TRAF6 (TNF receptor associated factor 6), IRF (interferon regulatory factor), and/or NF-κB (Akira, 2003; Takeda and Akira, 2004). These signals may lead to the production of various cytokines such as type I IFN (IFN-α and -1β) and proinflammatory cytokines (TNF-α, IL-1, IL-6, and IL-8; Akira, 2003; McGettrick and O’Neill, 2004; Takeda and Akira, 2004). Thus, the pathways are called “MyD88-dependent pathways” (Akira, 2003; McGettrick and O’Neill, 2004; Takeda and Akira, 2004). Signaling pathways fromTLR1, 2, 5–10 may be dependent on MyD88 (Akira, 2003; McGettrick and O’Neill, 2004; Takeda and Akira, 2004). However, TLR3 signaling pathways appear independent of MyD88 (Akira, 2003; McGettrick and O’Neill, 2004; Takeda and Akira, 2004). TLR4-mediated pathway may involve both MyD88-dependent and -independent pathways. With the exception of MyD88, four types of molecules in the cells have been confirmed as TIR domain-containing molecules, including TIRAP (TIR domain-containing adaptor protein), TRIF (TIR domain-containing adaptor including IFN-1β)/TICAM-1 (TIR domain containing adaptor molecule-1), TRAM (TIRF-related adaptor molecule), and SARM (sterile alpha motif and Armadillo motif domain-containing protein). Of these, TIRAP may be associated with MyD88, while IFN production by TRIF/TICAM-1 of TLR4 is independent of MyD88 (Akira, 2003; McGettrick and O’Neill, 2004; Takeda and Akira, 2004). These results suggest that the signaling pathways of each TLR are unique and complicated (Janssens and Beyaert, 2003; Akira et al., 2006). Nucleic acids (DNA or RNA) derived from pathogens induce the production of cytokines (Akira, 2003; Barbalat et al., 2011). Thus, TLRs can induce various cytokines against infections through activation of the signaling pathways. For example, TLR4 a ligand of F protein of RSV can activate MyD88-dependent signaling pathways resulting in the production of Th1 cytokines such as TNF-α, IL-6, IL-8, IL-12, and IL-18 (See and Wark, 2008; Klein Klouwenberg et al., 2009). On the other hand, TLR-3 activates MyD88 independent pathways leading to the production of IFN-1β (Akira, 2003; McGettrick and O’Neill, 2004; Takeda and Akira, 2004).

## NUCLEOTIDE-BINDING OLIGOMERIZATION DOMAIN FAMILY AND CYTOKINE PRODUCTION

In macrophages and epithelial cells, NLRs play a pivotal role in the recognition of bacteria and viruses as PRR molecules (**Figure 1**; Wells et al., 2011). At present, about 20 types of NLRs have been confirmed in humans (Schroder and Tschopp, 2010). The representative pathogen PRR-related NLRs are NLRP1, NLRP3 (cryopyrin), and NLRPC4 (Schroder and Tschopp, 2010). These molecules have both signal transduction domains in the N-terminal and leucine-rich repeats in the C-terminal (Schroder and Tschopp, 2010). Thus, NLRs show the properties of both PRR molecules and signaling molecules (Schroder and Tschopp, 2010). In addition, the N-terminal of NLRs acts as a caspase recruitment domain (CARD; Schroder and Tschopp, 2010). For example, NLRP3 binds pro-caspase-1 through activation of TLRs (TLR4) and forms “inflammasome” (Bauernfeind and Hornung, 2013). Activated NLRP3-pro-caspase-1 complex releases active caspase-1 (Schroder and Tschopp, 2010; Bauernfeind and Hornung, 2013). Active type caspase-1 activates pro-IL-1β and pro-IL-18, leading to their production in the cells (Schroder and Tschopp, 2010; Bauernfeind and Hornung, 2013).

## RETINOIC ACID-INDUCIBLE GENE-I LIKE RECEPTORS FAMILY

Retinoic acid-inducible gene-I and MDA5 (melanoma differentiation-associated protein 5) are localized in the cytosol and may be able to bind to some ssRNA viruses such as RSV, influenza virus, dengue fever viruses (DFV), and hepatitis C virus, leading to the production of type I IFN (IFN-α/β) in fibroblasts (Breiman et al., 2005; Loo et al., 2008; Jamaluddin et al., 2009; Bustos-Arriaga et al., 2011). In particular, it is known that RIG-I binds to ssRNA (5′-triphosphate RNA) derived from influenza virus and induces type I IFN (Loo et al., 2008). Furthermore, both RIG-I and MDA5 can bind to DFV type 2 genome and induce the production of type I IFN (Qin et al., 2011a). However, the roles of these molecules in innate immunity are not known at present.

## INFLAMMASOME, RLR-ASSOCIATED SIGNALING PATHWAYS, AND CYTOKINE PRODUCTION

Inflammasome as a PRR is a concept of the inflammatory reaction-associated protein complex (Schroder and Tschopp, 2010). It is suggested that both RIG-I and MDA5 can bind to an adaptor molecule, IPS-1(interferon-1β promoter stimulator 1), and activate NF-κB, resulting in the production of type I IFN (Schroder and Tschopp, 2010; Bauernfeind and Hornung, 2013). Inflammasome is composed of some protein complexes such as Apaf-1(apoptotic protease-activating factor 1), ASC (apoptosis-associated speck-like protein containing caspase recruitment domain), NOD (nucleotide-binding domain), and NALP (NACHT, LRR and PYD domain-containing protein; Schroder and Tschopp, 2010; Bauernfeind and Hornung, 2013). The complex recognizes various components of pathogens and uric acid as “danger signals” (Schroder and Tschopp, 2010; Bauernfeind and Hornung, 2013). After recognition of the signals, these signals activate ASC, leading to the conversion of procaspase-1 to caspase-1 (Schroder and Tschopp, 2010; Bauernfeind and Hornung, 2013). The protease caspase-1 activates proinflammatory cytokine precursors such as pro-IL-1β and pro-IL-18, leading to conversion to active forms of IL-1β and IL-18 (Schroder and Tschopp, 2010; Bauernfeind and Hornung, 2013). Interestingly, very recent studies suggest that various inflammatory diseases such as atherosclerosis and rheumatoid arthritis are associated with inflammasome, although the precise mechanisms are not known.

## RELATIONSHIPS BETWEEN PRRs, SIGNALING PATHWAYS, AND CYTOKINE PRODUCTION IN RESPIRATORY VIRUS-INFECTED CELLS

**FIGURE 4:**
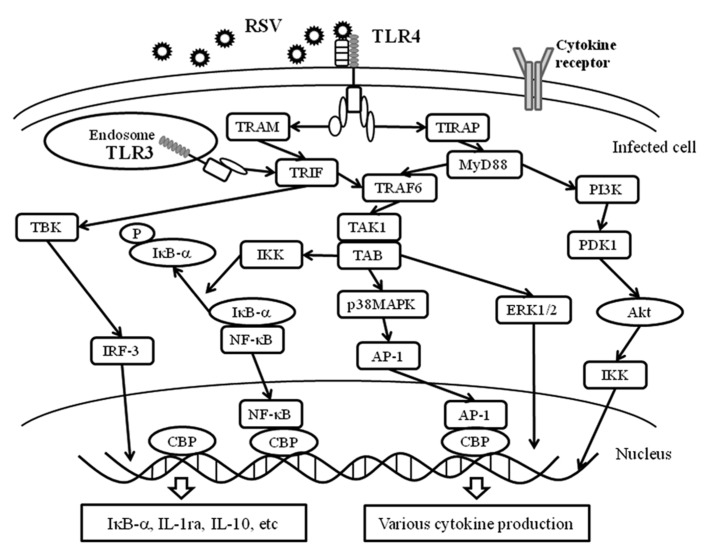
**PRRs and virus infection-associated signaling pathways.** The innate immune response to the fusion protein of an important respiratory pathogen of humans, RSV, is mediated by TLR4. The RSV F protein induces translocation of TLR4 to the endosome together with TRAM. TLR3 is expressed in intracellular endosomes and responds to the presence of double-stranded RNA (dsRNA), which forms as a product of the replication of the majority of RNA viruses such as RSV. TLR3 and TLR4 activate TRIF-dependent signaling, which activates NF-κB and IRF-3, and results in the induction of proinflammatory cytokines and type I IFNs. Moreover, TLR4 is able to signal via both MyD88-dependent and -independent pathways and is able to activate a response via IRF-3, NF-κB, AP-1, ERK and IKK. These receptors recognize RSV and induce an appropriate antiviral innate immune response in the infected cells. Akt, protein kinase B; ERK1/2, extracellular signal regulating kinase1/2; PDK1, phosphoinositide-dependent kinase 1; PI3K: phosphatidylinositol-3 kinase.

In general, cytokine production in immunological cells such as lymphocytes may be induced through each cytokine receptor on the cells (Salek-Ardakani and Croft, 2010; Rossol et al., 2011). Certainly, this process may occur in virus-infected cells (He and Greenberg, 2002). As mentioned previously, cytokine production may trigger innate immunity through PRRs including TLRs, RLRs, and inflammasomes (NLRPs-pro-caspase-1 complex; **Figure 1**; Yu and Levine, 2011). These receptors and/or intracellular protein complexes induce phosphorylation of the signaling molecules. Although the precise mechanisms are not known, the phosphorylation cascades of the molecules lead to cytokine production in virus-infected cells (Yu and Levine, 2011). The representative data of virus infection-associated signaling pathways is shown in **Figure 4**. Briefly, a previous report showed that RSV infection in human fetal lung fibroblasts (MRC-5 cells) induces various cytokines through the activation (phosphorylation) of Akt (murine thymoma viral oncogene homolog/protein kinase B), p38MAPK (mitogen activated protein kinase), ERK1/2 (extracellular signal-regulated kinase), and IκB-α (Seki et al., 2013). Human rhinovirus (HRV) infection in human bronchial epithelium cells (BEAS-2B cells) activated p38MAPK, ERK1/2, and NF-κB (nuclear factor kappa B protein). Human parainfluenza virus (HPIV) infection in MRC-5 cells activated p38MAPK and IκB-α (Yoshizumi et al., 2010). However, it is not currently known how PRRs are associated with the production of these cytokines.

## RESPIRATORY VIRUS INFECTION-ASSOCIATED CYTOKINE PRODUCTION

The summarized data of the cytokine production profiles in some respiratory virus-infected cells are shown in **Table 1**. Numerous reports show that most respiratory virus infections can induce the production of various types of cytokines *in vitro* and *in vivo* (Khaitov et al., 2009; Koetzler et al., 2009; Martínez et al., 2009; Sharma et al., 2009; Ishioka et al., 2011; Lewis et al., 2012; Seki et al., 2013). The findings of previous *in vitro* studies suggest that influenza virus type A [subtype A(H1N1) virus]-infected human airway epithelial cells produces significant amounts of IL-1, IL-6, and IL-8 (Hofmann et al., 1997). Production is associated with inflammasome (NLRP3-pro-caspase-1 complex; Pothlichet et al., 2013). HRV-infected airway epithelial cells produced IL-1, IL-6, IL-8, RANTES (regulated on activation normal T cell expressed and secreted), eotaxin, interferon-inducible protein (IP)-10, IL-11, TNF-α, granulocyte macrophage colony-stimulating factor (GM-CSF), IFN-1β, and IFN-λ (Yamaya, 2012). RSV-infected airway epithelial cells produced IL-1, IL-4, PIV-3, IL-6, RANTES, IL-8, IL-11, GM-CSF, andTNF-α (Yamaya, 2012). HPIV-3 infected human lung fibroblasts induced excessive expression of IL-1β, IL-1ra, IL-2, IL-4, IL-5, IL-6, IL-10, G-CSF, GM-CSF, IFN-γ, TNF-α, IL-8 IP-10, eotaxin, and RANTES (Yoshizumi et al., 2010).

**Table 1:** Cytokines and chemokines induced by virus infection.

Viruses	Samples and specimens	Cytokines and chemokines	Reference
RSV	A549	GM-CSF	Ishioka et al. (2011)
	MRC-5	IL-1β, IL-6, TNF-α, IL-1ra, IFN-γ, IFN-λ1a, IL-2, IL-12, IL-4, IL-5, IL-10, IL-13, G-CSF, GM-CSF, eotaxin, RANTES, IL-8, IP-10, MCP-1, MIP-1α, PDGF-bb, VEGF, FGF-basic	Seki et al. (2013)
	HEp-2	IL-1β, MCP-1, MIP-1α, RANTES	Martínez et al. (2009)
	Primary BECs	IFN-β, IFN-λ1	Khaitov et al. (2009)
HRV	Nasal aspirates	IFN-γ, IL-6, IL-8, IP-10, eotaxin, RANTES	Lewis et al. (2012)
	BEAS-2B	IL-6, TNF-α, IL-8, IP-10	Koetzler et al. (2009), Sharma et al. (2009)
PIV	MRC-5	IL-1β, IL-6, TNF-α, IL-1ra, IFN-γ, IL-2, IL-4, IL-5, IL-10, G-CSF, GM-CSF, eotaxin, RANTES, IL-8, IP-10, PDGF, VEGF	Yoshizumi et al. (2010)

*RSV, respiratory syncytial virus; HRV, human rhinovirus; PIV, parainfluenza virus.*

Previous *in vitro* studies have demonstrated that elevated IL-6, IL-8, and RANTES are found in sputum and serum in influenza virus infection (Yamaya, 2012). IL-6 and IL-8 were elevated in sputum and serum in HRV infection (Yamaya, 2012). Systemic avian influenza virus [subtype A(H5N1) virus] infection induced excessive production of proinflammatory cytokine, namely a cytokine storm (Ramos and Fernandez-Sesma, 2012). These results imply that cytokine production profiles may vary. Although the detailed information of the signaling pathways is not yet known, these differences may be associated with the pathophysiology of each respiratory virus infection (Schwarze and Mackenzie, 2013).

## RELATIONSHIP BETWEEN CYTOKINE PRODUCTION DUE TO RESPIRATORY VIRUS INFECTION AND THE PATHOPHYSIOLOGY OF VIRUS-INDUCED ASTHMA

Viral infections clearly induce inflammation at infected sites. A variety of complicated pathophysiological events occur at these sites. In broad terms, these events may constitute converged cell death and regeneration (Rennard and von Wachenfeldt, 2011). The process of events has been named “remodeling” (Al-Muhsen et al., 2011). Cytokines derived from respiratory virus infections may be associated with airway remodeling (Kuo et al., 2011). It is suggested that the major production sources of cytokines are airway epithelium, fibroblasts, myofibroblasts, and leukocytes within infected regions (Westergren-Thorsson et al., 2010). These cytokines may be associated with remodeling processes following respiratory virus infections (Holtzman et al., 2002).

## CONCLUSION

Since the discovery of PRRs, remarkable progress has been made toward understanding the role of innate immunity against pathogens. However, the precise roles of PRRs, the mechanisms of intrinsic signaling pathways, and cytokine production with regard to PRRs are not fully understood. In addition, recent studies suggest that PRRs may be associated with various inflammatory diseases such as gout, rheumatoid arthritis, and atherosclerosis. It would be beneficial to clarify the functional relevancy of infectious diseases and other inflammatory diseases in the near future.

## Conflict of Interest Statement

The authors declare that the research was conducted in the absence of any commercial or financial relationships that could be construed as a potential conflict of interest.
